# Motor neuron-derived Thsd7a is essential for zebrafish vascular development via the Notch-dll4 signaling pathway

**DOI:** 10.1186/s12929-016-0277-9

**Published:** 2016-08-02

**Authors:** Lawrence Yu-Min Liu, Min-Hsuan Lin, Zih-Yin Lai, Jie-Peng Jiang, Yi-Ching Huang, Li-En Jao, Yung-Jen Chuang

**Affiliations:** 1 Department of Medical Science & Institute of Bioinformatics and Structural Biology, National Tsing Hua University, Hsinchu, 30013 Taiwan; 2 Division of Cardiology, Department of Internal Medicine, Mackay Memorial Hospital Hsinchu Branch, Hsinchu, 30071 Taiwan; 3 Department of Cell Biology and Human Anatomy, UC Davis School of Medicine, 4415 Tupper Hall, One Shields Avenue, Davis, CA 95616 USA

**Keywords:** Angiogenesis, Neurogenesis, Neurovascular interactions, Thsd7a, Notch

## Abstract

**Background:**

Development of neural and vascular systems displays astonishing similarities among vertebrates. This parallelism is under a precise control of complex guidance signals and neurovascular interactions. Previously, our group identified a highly conserved neural protein called thrombospondin type I domain containing 7A (THSD7A). Soluble THSD7A promoted and guided endothelial cell migration, tube formation and sprouting. In addition, we showed that *thsd7a* could be detected in the nervous system and was required for intersegmental vessels (ISV) patterning during zebrafish development. However, the exact origin of THSD7A and its effect on neurovascular interaction remains unclear.

**Results:**

In this study, we discovered that zebrafish *thsd7a* was expressed in the primary motor neurons. Knockdown of Thsd7a disrupted normal primary motor neuron formation and ISV sprouting in the *Tg(kdr:EGFP/mnx1:TagRFP)* double transgenic zebrafish. Interestingly, we found that Thsd7a morphants displayed distinct phenotypes that are very similar to the loss of Notch-delta like 4 (dll4) signaling. Transcript profiling further revealed that expression levels of *notch1b* and its downstream targets, *vegfr2/3* and *nrarpb*, were down-regulated in the Thsd7a morphants. These data supported that zebrafish Thsd7a could regulate angiogenic sprouting via Notch-dll4 signaling during development.

**Conclusions:**

Our results suggested that motor neuron-derived Thsd7a plays a significant role in neurovascular interactions. Thsd7a could regulate ISV angiogenesis via Notch-dll4 signaling. Thus, Thsd7a is a potent angioneurin involved in the development of both neural and vascular systems.

**Electronic supplementary material:**

The online version of this article (doi:10.1186/s12929-016-0277-9) contains supplementary material, which is available to authorized users.

## Background

Development of neural and vascular systems displays high similarity and this congruence is precisely regulated by complex neurovascular interactions and common guidance cues. The blood vessels supply nutrients, oxygen and growth factors to stimulate the proliferation and differentiation of neural cells. Concurrently, the neural cells provide guidance cues to direct endothelial cells to migrate and expand toward the neural tissues in need [1, 2]. There have been reports indicating that neurogenesis occurs prior to angiogenesis in order for the developing neurons to acquire nutrition to sustain growth [3–5]. Signal molecules such as ephrins, semaphorins, slits, and netrins [6–8] that have dual functions on guiding neurogenesis and angiogenesis are thus named “angioneurins” [9]. These angioneurins regulate vascular and nervous differentiation, proliferation, survival and migration during development.

In our previous study, we found a novel conserved protein, Thrombospondin type-I domain containing 7a (THSD7A), is highly expressed in human placental vasculature and umbilical vein endothelial cells (HUVECs). Overexpression of THSD7A in HUVECs inhibits directed cell migration and tube formation, while suppression of THSD7A generates the opposite effects. A soluble form of THSD7A increases the number of new vessel branching points, promotes HUVECs migration and tube formation during angiogenesis via a FAK-dependent mechanism [10]. Moreover, we observed that zebrafish ortholog of THSD7A transcript is detected in the central nervous system along the ventral edge of neural tube and on the growth path of intersegmental vessels (ISV). MO knockdown of Thsd7a disrupts the ISVs patterning in zebrafish embryos. Collectively, our previous findings suggest that zebrafish Thsd7a is a novel angioneurin candidate derived from neuron cells to regulate angiogenesis [10–12]. In the present study, we further confirmed the expression of Thsd7a in primary motor neurons by generating a transgenic *Tg(thsd7a:GFP)* zebrafish line and characterized Thsd7a’s essential function in neurovascular interactions. The possible signal pathways involved in Thsd7a-mediated angiogenesis and motor neuron development were also explored and investigated.

## Methods

### Zebrafish care

Wild-type AB strain, *Tg(kdr:EGFP)*, and *Tg(mnx1:TagRFP)* transgenic strains were used [13]. Zebrafish embryo was incubated at 28.5 °C and staged as described [14].

### Construction of the *thsd7a-GFP* BAC transgenic zebrafish

*thsd7a-GFP* transgenic zebrafish was created by using bacterial artificial chromosome (BAC) homologous recombination. The *thsd7a*-containing BAC clone was purchased commercially (Plasmid HUKGB735L10208Q, Source BioScience). First, *thsd7a* BAC plasmid was extracted from overnight culture cell broth by Midiprep kit (Invitrogen, Carlsbad, CA). The extracted BAC was transformed into EL250 competent cells by electroporation and the flip recombinase activity was induced by 42 °C incubation, BAC-contained EL250 competent cells were selected with chloramphenicol antibiotic. Secondly, specific forward primer was designed by adding 45 base pairs of gene specific sequence together with the green fluorescent protein (GFP) forward primer, and 45 bp of gene specific sequence together with the anti-kanamycin reverse primer. We used a PCR long tailing method to make the GFP-Km DNA cassette for insertion of GFP into the *thsd7a* BAC clone. After homologous recombination, the first exon of *thsd7a* was partially replaced with GFP, resulting in the *thsd7a-GFP* construct driven by the *thsd7a* regulatory elements in the BAC clone. The construct was then microinjected into zebrafish embryos at one to two cell stages to generate stable lines.

### Morpholino microinjection and mRNA rescue

Morpholino phosphorodiamidate oligonucleotides (morpholino, MO) were synthesized by Gene Tools (Philomath, OR) to target splice junctions of the zebrafish *thsd7a* gene. The MO sequences were as follows: MO1, 5′-TGTATGTTTTTACCCACCATGACTG-3′; 5-base mismatch control for MO1 (msMO1), 5′-TCTATCTTTTTAGCCACGATGAGTG-3′; MO2, 5′-GTGCCA GTTTTGTTACCGTCTTTGC-3′; 5-base mismatch control for MO2 (msMO2), 5′-GTCCCACTTTTCTTACGGTCTTTCC-3′. The injection dosage used is 2 ng of MO1 and 9 ng of MO2 to each embryo. *Thsd7a* mRNA was synthesized using the mMESSAGE mMACHINE system (Ambion, Autstin, TX) with SP6 RNA polymerase. Murine Notch gene homolog 1 (Notch1) was cloned downstream of the CMV promoter in the pTCN vector (BC138442; constructed by transOMIC. Huntsville, AL). Co-injection of MO1 with 0.4 ng of *thsd7a* mRNA or 12.5 pg of *notch1* construct into zebrafish embryos were performed at one-cell stage of development.

### Whole-mount in situ hybridization

Embryos were fixed in 4 % paraformaldehyde overnight at 4 °C and washed by 1X phosphate buffered saline tween-20 (PBST). They were then treated with protease K for 25 min and refixed in 4 % paraformaldehyde for 20 min at room temperature. The embryos were soaked in hybridization buffer (Hyb) at 65 °C for 3 h before the specific probe was added to the embryos in Hyb buffer overnight at 65 °C. The embryos were then washed with 75 % Hyb/25 % 2X SSC, 50 % Hyb50 %/2X SSC, and 25 % Hyb/75 % 2X SSC each at 65 °C for 10 min, then in 0.2X SSC twice for 1 h. After blocking with 2 % bovine serum albumin and goat serum in maleic acid buffer at room temperature for 3 h, AP-conjugated anti-DIG antibody was added into the blocking buffer overnight at 4 °C. The embryos were washed with maleic acid buffer four times at room temperature for 30 min, before they were treated with the NBT/BCIP substrate (Roche, Basel, Switzerland) to react at room temperature for 3 h. Images were taken by using a stereomicroscope (SMZ1500; Nikon, Kanagawa, Japan) equipped with a CCD camera (DS-Fi1; Nikon, Kanagawa, Japan) and Imagepro plus AMS software (Media Cybernetics, Bethesda, MD).

### Immunofluorescence staining

Embryos were fixed in 4 % paraformaldehyde overnight at 4 °C followed by washing with 1X PBST 10 min for 3 times at room temperature, and then permeabilized with ice cold acetone 30 min at 4 °C. After washing with maleic acid buffer (containing 150 mM maleic acid, 100 mM NaCl, pH7.5) 3 times, 10 min each, embryos were blocked with the blocking reagent (containing 2 % goat serum and 2 % BSA in malice acid buffer) for two hours at room temperature. The primary antibody was then added to the blocking reagent and incubated at 4 °C overnight. Finally, embryos were washed with maleic acid buffer 4 times, 30 min each followed by adding appropriate secondary antibody and incubating for 2 h at room temperature. After embryos were washed with maleic acid buffer 4 times, 30 min each again, the embryos were mounted and imaged by a confocal microscope (A1R; Nikon, Kanagawa, Japan). The rabbit anti-EGFP antibody was from Novus and used in 1:600 dilution. The mouse anti-zebrafish Znp-1 antibody was from Zebrafish International Resource Center and used in 1:50 dilution. The goat anti-rabbit IgG Dylight 488 and goat anti-mouse IgG Dylight 549 antibodies were all from Jackson and used in 1:400 dilution.

### Real time-quantitative PCR analysis

RNA samples were extracted at 48 h post fertilization (hpf) for morpholino-injected and non-injected zebrafish embryos. cDNA was synthesized by using Transcriptor First Strand cDNA Synthesis kit (Roche, Basel, Switzerland). The specific transcripts of zebrafish were amplified by PCR (Primer sequences are listed in Additional file 1: Table S1). The RT-qPCR reaction was performed using SYBR Green Master Mix (Applied Biosystems, Carlsbad, CA) according to the manufacturer’s instructions and data were analyzed using ABI 7500 System SDS Software. All RT-qPCR products were cloned into the pCRII-TOPO vector (Invitrogen, Carlsbad, CA) and sequenced directly.

### Statistics

Student’s *t*-test (with two-tailed distribution and unequal variance) was performed in Microsoft Excel to test for differences between two sample populations.

## Results

### Expression of Zebrafish *thsd7a* in primary motor neurons

**Fig. 1:**
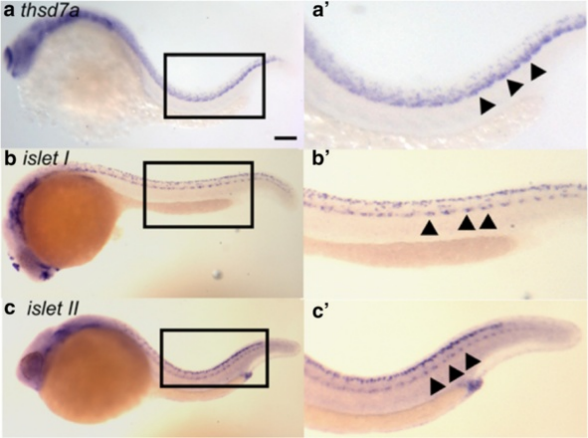
*thsd7a* was first expressed in primary motor neurons. Whole-mount ISH showed the spatiotemporal expression pattern of *thsd7a, islet I and islet II* at 24 hpf in wild type zebrafish embryos. Anterior is to the left. a: *thsd7a* transcripts were detected along the ventral edge of the neuron tube indicated by arrowheads. b, c: *islet I* (MiP and RoP marker) and *islet II* (CaP marker) were expressed in primary motor neurons along the ventral edge of the neuron tube indicated by arrowheads. Rohon-Beard sensory neurons can be seen along the dorsal edge of the neuron tube. a’, b’, c’: Enlarged images of the boxed regions in panel a, b and c, respectively. Scale bar is 100 μm

Our previous study suggested that zebrafish *thsd7a* is expressed in the central nervous system (Fig. 1a-a’), likely in the neurons or glial cells [12]. To precisely identify the cells expressing *thsd7a*, we compared its expression pattern with markers of neuronal cells by in situ hybridization (ISH) (Additional file 2: Figure S1). We found that the spatiotemporal expression patterns of *islet I* and *islet II* transcripts were also detected along the ventral edge of the neuron tube with discontinuous clusters, which were similar to that of *thsd7a*. Islet I is a middle primary motor neuron (MiP) and rostral primary motor neuron (RoP) marker, and islet II is a caudal primary motor neuron (CaP) marker. (Fig. 1b-c’). Both are specific markers for primary motor neurons [15]. These results indicated that zebrafish *thsd7a* was likely expressed in the primary motor neurons during development.

### Generation of *thsd7a:*GFP transgenic zebrafish

**Fig. 2:**
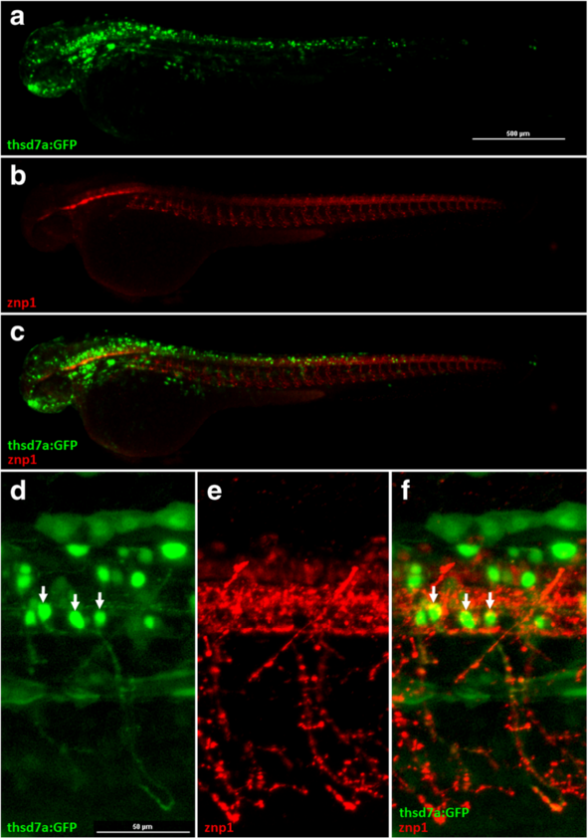
The signals of *Tg(thsd7a:GFP)* were detected in primary motor neurons. Representative images of the *Tg(thsd7a:GFP)* transgenic zebrafish at 48 hpf. Thsd7a and motor neurons were shown in green and red, respectively. Anterior is to the left. **a**-**c** GFP signals driven by *thsd7a* promoter were detected in the brain and the neural tube, consistent with ISH results. Moreover, the signals at the neural tube co-localized with anti-Znp1 antibody (shown in *red*). **d**-**f** Enlarged images of the neural tube showed GFP signals were expressed in primary motor neurons, indicated by arrows

To further confirm the finding that *thsd7a* was expressed in primary motor neurons, we generated a transgenic zebrafish *Tg(thsd7a:GFP)* with the expression of green fluorescent protein (GFP) driven by *thsd7a* regulatory elements. At first, we compared the sequences of human and mouse *thsd7a* promoters by Cartwheel genomic sequence analysis. We discovered a highly conserved region between the human and the mouse, which was located about 3 kilobases (Kb) upstream of the translation start site. We further found that a similar conserved region is also present about 3 kb upstream of the zebrafish Thsd7a ortholog. We used PCR reaction to amplify a GFP-Km cassette that was flanked by target homologous BAC sequences located between the *thsd7a* translational start site and the first exon (Additional file 3: Figure S2). After homologous recombination, the final constructs were isolated, and microinjected into wild-type AB zebrafish embryos at one cell stage. At 48 hpf, we detected the GFP signals in the central nervous system (Fig. 2a-c). The GFP expression pattern driven by *thsd7a* regulatory elements was consistent with that detected by *thsd7a* ISH. When the *Tg(thsd7a:GFP)* zebrafish were probed with the Znp1 antibody, a specific marker for primary CaP motor neurons, we observed an extensive overlap between the GFP and the Znp1 signals (Fig. 2d-f). This result indicated that *thsd7a* is expressed in primary motor neurons.

### Zebrafish Thsd7a is essential for angiogenesis and motor neuron formation

**Fig. 3:**
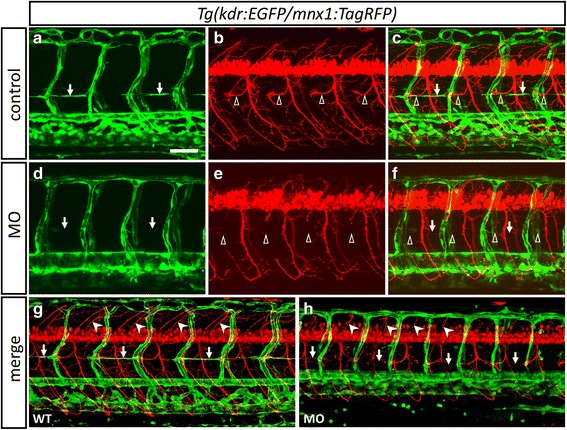
Loss of *thsd7a* impaired PAC, RoP axons and associated secondary motor neuron axons formation. Representative images showed the effect of Thsd7a knockdown on PAC angiogenesis and RoP neurogenesis by morpholino in *Tg(kdr:EGFP/mnx1:TagRFP)*. Embryos were examined at 48 hpf (**a**-**f**) and 78 hpf (**g**-**h**). Anterior is to the left. **a**-**c**, **g**: Uninjected control. **d**-**f**, H: Embryos were injected with MO1 and MO2. Vasculature and motor neuron shown in green and red, respectively. PAC, RoP and MiP are indicated by arrows, open arrowheads and arrowheads, respectively. **a**, **b**: PAC and ROP axons were formed normally along HMS in control group. **d**: PAC was completely absent at HMS in morphants. **e**: RoP axons were absent at HMS. Moreover, MiP axons and some secondary motoneuron grew aberrantly. **c**, **f**: Merged images of panel **a**, **b** and **d**, **e** respectively. **g**: PAC and primary motoneurons grew normally. **h**: Some initially absent RoP axons reappeared but their trajectory were slightly shifted and PAC still absent. MiP axons were shorter with abberant outgrowth. Scale bar is 100 μm

We have previously showed that zebrafish *thsd7a* is required for ISV angiogenic patterning [12]. To test whether Thsd7a also participates in neurovascular interactions, we examined the role of Thsd7a in motor neuron guidance and development. We knocked down Thsd7a by antisense morpholinos in the *Tg(kdr:EGFP/mnx1:TagRFP)* double-transgenic embryos. At 48 h post fertilization (hpf), the Thsd7a morphants showed a complete loss of parachordal chain (PAC) in the developing vasculature along the horizontal myoseptum (HMS) (Fig. 3a vs. d). Intriguingly, the same morphants also showed the lack of laterally projecting motor axons at the HMS derived from the RoP primary and secondary motor neurons. An aberrant outgrowth of the dorsally projecting axons could also be observed (Fig. 3b vs. e). Similar findings could also be found in the interupted outgrowth of CaP axons identified by Znp-1 staining at 55 hpf. These defects in the PAC loss and motor axon outgrowth were not observed in the morphants injected with the control mismatched morpholinos (Fig. 3c vs. f). At later stages (e.g., 78 hpf), laterally projecting motor axons near the HMS appeared in some of the somite segments, but they showed abberant branching. Dorsally projecting motor axons in these morphants continued to exhibit irregular patterns with shorten axons and abberant outgrowth (Fig. 3g vs. h). In contrast, the PAC in the developing vasculature remained absent at later stages (Fig. 3h).

### Thsd7a knockdown affected Notch1b expression

Previously, we found that knockdown of Thsd7a caused a delay in ISV growth in zebrafish embryos. Upon close examination, we found that the endothelial tip cells on the angiogenic ISV displayed multiple filopodia expansions, arranged in fan-shape morphology, which is a sign of outgrowth disorientation during angiogenic pathfinding (Additional file 4: Figure S3). These abnormalities might cause the angiogenic sprouts to form aberrant connection to adjacent vessels in ISV, SIV and CtA networks. Together, these phenotypes are very similar to the phenotypes observed under the loss of Notch-dll4 signaling pathway [16–18].

Notch-dll4 signaling pathway plays an important role in mediating the tip and stalk cell induction at the angiogenic spout [19, 20]. Notch1b is an isoform of notch receptor in zebrafish that works with dll4 to regulate the proliferation and migration of intersomitic vessel endothelial cells [21]. It has been shown that notch1b is a crucial receptor in the stalk cell of angiogenic sprout, which acts to communicate with the tip cells through dll4 [22]. Knockdown of notch1b results in hyper-branching of ISV during angiogenesis [18].

**Fig. 4:**
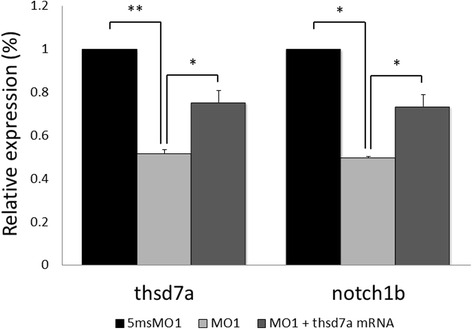
Knockdown of Thsd7a affected expression of genes related to Notch/dll4 signaling pathway. MO1 and 5msMO1 treated embryos at 48 hpf were collected for quantitative real-time PCR. *thsd7a* expression was down-regulated by 49 %. Notch1b was significantly downregulated by Thsd7a knockdown. A 51 % decrease of notch1b expression was observed in the Thsd7a morphants as compared with 5msMO1. Co-injection of MO1 and *thsd7a* mRNA increased both *thsd7a* and *notch1b* expression levels by 24 %. Endogenous *β-actin* was used as an internal control. Each experiment was performed in triplicate and repeated at least three times. *, *P* < 0.05; **, *P* < 0.01; Student’s *t*-test. The error bars shown mean ± SEM

Based on these reports, we investigated whether Thsd7a is involved in the notch-dll4 signaling pathway to regulate angiogenic sprouting during development. We started by real-time quantitative PCR assays and ISH analysis to analyze the expression profiles of *thsd7a, notch1b* and *dll4* in 48 hpf Thsd7a morphants and the control embryos. After receiving Thsd7a morpholinos, the expression of *thsd7a* was decreased by 49 % as compared with that in the control morphants. We also observed 51 % decrease of *notch1b* expression in the Thsd7a morphants (Fig. 4), whereas the expression level of *dll4* showed no significant difference (data not shown). Co-injection of 2 ng MO1 and 0.4 ng of *thsd7a* mRNA signficantly increased the expression levels of *thsd7a* and *notch1b* mRNA. To further establish their relationship, we rescued Thsd7a-knockdown phenotype with *notch1* mRNA. The penetrance of the MO1-induced ISV defect was 74.89 %, which was consistent with our previous findings. Co-injection of 2 ng MO1 and 12.5 pg *notch1* mRNA resulted in a penetrance of 58.51 %, which was lower than that caused by injection of 2 ng MO1 alone. In addition, notch1 rescue significantly increased the normal ISV phenotype from 25.11 to 36.14 % (*p* = 0.026) (Table 1). These data suggested that Thsd7a may be an upstream regulator of notch1 during development. It is also possible that notch1 may mediate Thsd7a effect in regulating ISV development.

**Table 1:** Phenotype frequencies of ISV defects in embryos at 48 hpf^a^

Injection	Normal ISV (%)	Aberrant ISV (%)	Notch1-induced deformity (%)	Total live embryos (experiment repeats)
5msMO1				
2 ng	67 ± 14	33 ± 15	0 ± 0	230 (3)
MO1				
2 ng	25 ± 1	75 ± 2	0 ± 0	217 (3)
MO1/Notch1				
2 ng/12.5 pg	36 ± 1	59 ± 4	5 ± 3	312 (3)

Mean ± SEM was estimated from experimental replicates

*ISV* intersegmental vessel, *MO* morpholino, *hpf* hour post fertilization

^a^Frequency was calculated as the number of embryos with the indicated phenotype divided by the number of live embryos in each experiment

**Fig. 5:**
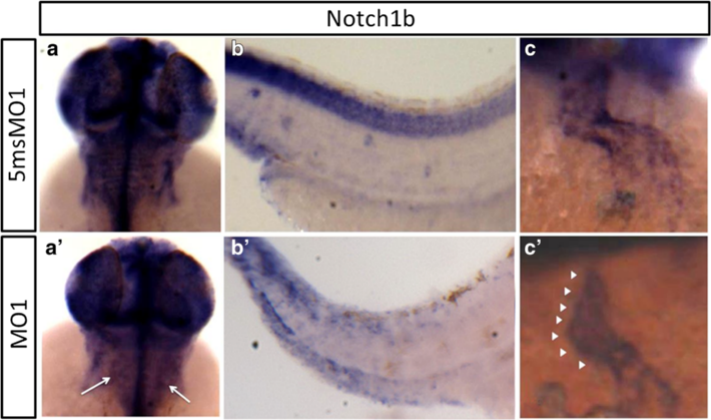
Thsd7a knockdown resulted in aberrant Notch1b expression pattern. Whole-mount ISH revealed the spatiotemporal expression patterns of notch1b at 48 hpf with either Thsd7a MO1 (a’-c’) or 5msMO1 (a-c). In the control group, notch1b transcripts were detected in the hind brain with a grid-like pattern (a). In comparison, Thsd7a morphants lost the grid-like expression pattern of notch1b (a’), which were indicated by the white arrows. Reduced expression of notch1b in the spinal cord (b’) was observed. In Thsd7a morphants, notch1b ectopic expression was observed throughout the endocardium in the heart tube (c’) indicated by white arrow head

We also performed ISH experiments to compare the expression patterns of *notch1b* between Thsd7a morphants and the corresponding control morphants. The expression pattern of *notch1b* in the head of Thsd7a morphants lost the grid-like pattern in the hindbrain (Fig. 5a, a’). This grid-like structure in the hindbrain of zebrafish embryos represents successive segments, which contain three types of repeated reticulospinal neurons. These reticulospinal neuron cells are thought to mediate the escape response for zebrafish to avoid predators [23]. In the spinal cord of Thsd7a morphants, we observed expression levels of *notch1b* were clearly reduced when compared with controls (Fig. 5b, b’), implicating a possible down-regulation of *notch1b* expression in this region when Thsd7a was knocked down.

Furthermore, in the embryonic heart of Thsd7a morphants, we observed apparent ectopic expression of *notch1b* throughout the heart tube, whereas *notch1b* expression in the heart of the control morphants had a preferential expression in the AV canal (Fig. 5c, c’). Thsd7a morphant heart displayed significant looping defect and malformation of the AV cancal (data not shown). These data are in agreement with a previous report that notch1b is involved in the specification of central cardiac conduction tissue [24].

Taken together, our data strongly suggested that loss of Thsd7a expression would reduce the expression level and disrupt the expression pattern of *notch1b*. This misregulation of notch1b expression would lead to the malformation of the AV canal and significant vascular anomaly with hyper-branching of SIV, CtA and ISV in developing zebrafish.

### Thsd7a knockdown affected the expressions of endothelial tip and stalk cell markers

**Fig. 6:**
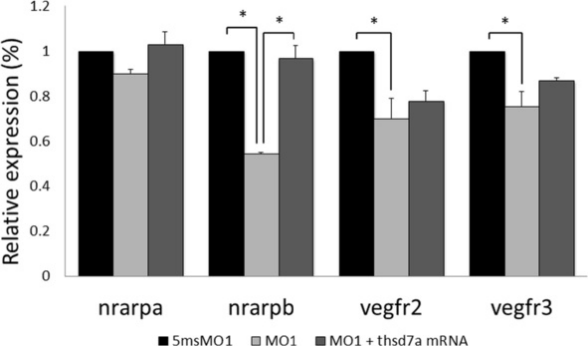
Expression of endothelial tip and stalk cell markers during angiogenesis was affected by Thsd7a knockdown. MO1 and 5msMO1 treated embryos at 48 hpf for quantitative real-time PCR. The expression level of stalk cell marker, *nrarpb*, was decreased by 54 % in Thsd7a morphants, though unchanged in *nrarpa*. The tip cell markers, *vegfr2* and *vegfr3*, were decreased by 30 and 25 %, respectively. Co-injection of MO1 and *thsd7a* mRNA signficantly rescued nrarpb expression level. Endogenous *β-actin* was used as an internal control. Each experiment was performed in triplicate and repeated at least three times. *, *P* < 0.05; Student’s *t*-test. Error bars shown mean ± SEM

Notch1 signaling regulates endothelial tip cell formation during angiogenesis [19]. To test whether loss of Thsd7a would impair angiogenesis due to the failure of communication between tip and stalk cells, we examined tip cell marker *vegfr2/3* and stalk cell marker *nrarpa/b* [25] by quantitative RT-PCR. We found that the expression levels of *vegfr2* and *vegfr3* were decreased by 30 and 25 %, respectively in the Thsd7a morphants. Morphologically, we observed the misdirection of the endothelial cell outgrown and migration. Furthermore, we observed 54 % reduction in the expression level of *nrarpb* in the Thsd7a morphants (Fig. 6). Co-injection of 2 ng MO1 and 0.4 ng of *thsd7a* mRNA successfully rescued the reduction of *nrarpb* expression level. Nrarp is a downstream molecule of the Notch signaling pathway [26] and regulates stalk cell proliferation and stablizes new endothelial connections during angiogenesis [25]. Together, these results suggested that Thsd7a in zebrafish regulates the tip cell orientation at the growing angiogenic sprouts through the regulation of the Notch signaling.

## Discussion and conclusion

Our present study showed that zebrafish Thsd7a is a motor neuron-derived protein essential for both neurogenesis and angiogenesis during zebrafish embryonic development. Using the a transgenic reporter approach, we observed that *thsd7a* expression was co-localized with the expression of motor neurons in the central nervous system, including the midbrain, hindbrain, cerebellum, telencephalon and spinal cord. In situ hybridization assays also demonstrated that *thsd7a* was expressed in the primary motor neurons. Loss-of-function analysis of Thsd7a also revealed that Thsd7a plays a critical role in maintaining the fan-shape morphology of the endothelial tip cells of the intersegment vessels and that Thsd7a is required for the outgrowth of motor neuron axons. In addition, Thsd7a is also required for the formation of the parachordal chain (PAC), the precursors of the zebrafish lymphatic vessels that share the same growth path of the RoP axons.

Angioneurins are either secreted or transmembrane proteins, and exert their function by regulating the adhesion, differentiation, survival, proliferation and migration of principal component cells of the vascular and nervous systems [8, 27, 28]. In our previous study [10], we reported that THSD7A contains several thrombospondin type 1 repeats (TSRs) that have been shown to be a potent regulator of angiogenesis in vivo. Interestingly, some of the angioneurins with TSRs are also shown to be expressed in the developing nervous system [29]. Human THSD7A is a membrane-associated N-glycoprotein with a possible soluble form, which could be released into the extracellular space. Consequently, the secreted form of THSD7A can promote endothelial cell migration during angiogenesis and increase the number of new vessel branching points. This is consistent with the finding that knocking down of Thsd7a leads to a delayed growth of intersegmental vessels and abnormal branching in zebrafish embryos, which can be partially rescued by *thsd7a* mRNA and transplantation of wild-type cells [12]. Taken together, our study suggests that zebrafish Thsd7a is a novel angioneurin derived from motor neurons. It plays critical roles in guiding the neurovascular development, as the roles of other angioneurins such as slit, semaphorins, ephrins, and netrins [3].

A recent study has identified THSD7A as an autoantigen involved in adult idiopahtic membranous nephropathy [30]. THSD7A is found to be expressed in podocytes, rather than in glomerular endothelial cells, and likely forms an in situ immune complex, resulting in THSD7A-associated membranous nephropathy. This finding coincides with our data in zebrafish that Thsd7a is expressed in perivascular cells that regulate angiogenesis. Podocytes are known to mediate glomerular endothelial cell angiogenesis via VEGF-A and Ang-1 pathways [31]. However, whether Thsd7a functions as an angiogenic factor during human glomerular development is yet to be studied. In addition, it is unclear if circulating soluble Thsd7a is present as our previous data suggest in these patients.

In the present study, we found Thsd7a could influence tip cell pathfinding in addition to vascular patterning. Vegfr2 and Vegfr3 were significantly down-regulated when Thsd7a was knocked down. The loss of Vegfr2 and Vegfr3 could prevent the endothelial tip cells from receiving VEGF signals and disrupt the endothelial cell pathfinding during angiogenesis, which is consistent with our observation that the tip cells adapted a fan-shape morphology and were trapped in the early stage of migration process. As a result, the tip cells extended pseudopodia to make contact with adjacent vessels and formed hyper-branching among the angiogenic vessels, likely because the tip cells of the affected angiogenic vessels failed to sense the growth factors in the immediate microenvironment. Moreover, Vegfc/Vegfr3 signaling is also important in maintainance of motor neurons and alignment of axonal growth with dorsal aorta [32]. Similar observations are also noted when the Netrin/Unc5 pathway is perturbed in a previous zebrafish-based study [33]. It is noteworthy that both Thsd7a and Netrin1a are involved in the FAK signaling pathway [34]. Netrin attracts motor neuron axons, which subsequently direct endothelial cell migration and PAC formation. However, the potential role of Thsd7a in the Netrin1a signaling pathway awaits further investigation.

Notch signaling pathway has been implicated in the regulation of both angiogenesis and neurogenesis. We observed that the level of Notch1b was significantly downregulated in the Thsd7a morphants. This downregulation of Notch1b level likely contributes to the abnormal sprouting and branching during angiogenesis. In the absence of Notch downstream signaling factors, zebrafish embryos display excessive filopodia activity during angiogenesis, marked by increased tip cell numbers and enhanced endothelial cell migration at the angiogenic sprouts [20]. This is similar to what we observed in the Thsd7a morphants, which displayed unrestrained filopodia activity in endothelial tip cells as well. We also showed that Notch1b was expressed in the hindbrain and spinal cord of zebrafish embryo, supporting the role of Notch signaling during central nervous system development. Previous zebrafish studies reported that Notch signaling is required to maintain proliferative neural precursors and regulate neuronal differentiation [35, 36]. Significantly, this notch1b expression was lost upon Thsd7a knockdown.

**Fig. 7:**
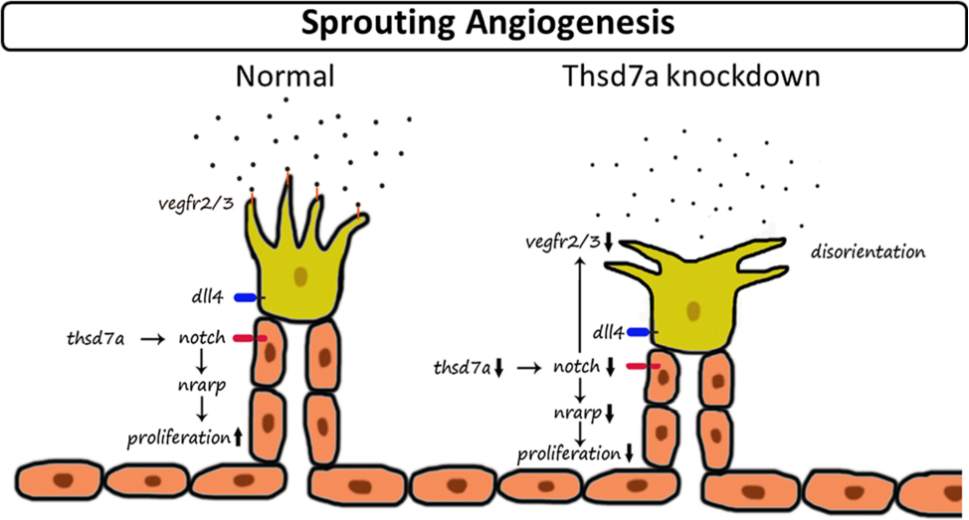
Proposed molecular mechanism of Thsd7a on tip and stalk. Our data suggest that Thsd7a could down-regulate notch expression to affect tip and stalk cell communication. Moreover, the down-regulation of vegfr2/3 on tip cell results in the loss of guidance cues and growth path disorientation and the down-regulation of nrarp leads to decreased endothelial cell proliferation and irregular vessel patterning. Taken together, this model might explain why Thsd7a knockdown results in the hyper-branching of angiogenic sprout

During angiogenesis, Notch signaling induces the expression of nrarp in the stalk cells, which regulates vasculature density by controlling stalk cell proliferation and maintains the vessel stability by stabilizing new endothelial connections [25]. In addition, the reduction of Notch signaling in endothelial stalk cells will initiate the formation of new endothelial tip cells [17, 19, 21]. These observations are consistent with our findings that excessive vessel branching occurred in the subintestinal vessels and central artery when Thsd7a was knocked down. The data elucidate why the new blood vessels cannot grow along the right path, which leads to aberrant connection between adjacent vessels during angiogenesis (Fig. 7). Moreover, these abberant vascular growth are partially rescued by co-injection of Thsd7a or Notch1 mRNA, reflecting the complex interactions between Notch and VEGF signaling pathways during angiogenesis [37]. Thus, our data suggest that Thsd7a-Notch signal pathway is important in regulating angiogenic vessel patterning and neuron outgrowth. However, the exact mechanism on how Thsd7a regulates Notch1b during neurovascular interactions remains to be elucidated.

In conclusion, we have provided new evidence to support the notion that Thsd7a is a potent angioneurin with a critical role in regulating not only angiogenesis, but also motor neuron development. We suggest that Thsd7a functions through interactions with the Notch-dll4 signaling in neurovascular interactions. Understanding how Thsd7a influences the development of vascular and nervous systems will greatly expand our knowledge on the neurovascular interaction and will shed lights on novel therapeutic means to treat diseases linked to the dysfunction of these two parallel systems.

## Abbreviations

BAC, bacterial artificial chromosome; CaP, caudal primary motoneuron; CtA, central artery; Dll4, delta like 4; HMS, horizontal myoseptum; Hpf, hours post fertilization; HUVEC, human umbilical vein endothelial cells; Hyb, hybridization buffer; ISH, In situ hybridization; ISV, intersegmental vessels; MiP, middle primary motoneuron; MO, morpholino; NICD, notch intracellular domain; PAC, parachordal chain; PBST, phosphate buffered saline tween-20; RoP, rostral primary motoneuron; RTq-PCR, real time-quantitative PCR analysis; SIV, subintestinal vessels; SSC, sodium citrate buffer; THSD7A, thrombospondin type I domain containing 7A

## Additional files

Additional file 1: Table S1. Primer list of Real Time-quantitative PCR analysis. (PNG 561 kb) Additional file 2: Figure S1. Comprehensive in situ hybridization analysis of *thsd7a* expression to different neuronal markers. The expression of different neuronal markers include nestin (progenitor cells of nervous system)、olig2 (oligodendrocyte)、gfap (glial fibrillary acidic protein; astrocyte)、huc (Hu antigen C; mature neuron)、islet I (MiP and RoP motoneuron)、islet II (CaP motoneuron) and netrin1a (neuron tube and HMS). (PNG 210 kb) Additional file 3: Figure S2. The approach of construct *thsd7a* transgenic zebrafish. Representative image is the flow chart of making transgenic fish construct. PCR amplified cassette containing GFP was flanked by target homologous BAC sequence indicated by gray box. The target homologous site was designed to locate between the *thsd7a* translational start site and the first exon. BAC containing PCR product and *thsd7a* promoter were then electroporated into EL250 competent cells which can induce homologous recombination activity. The end product was microinjected into zebrafish embryos at one-to-two cell stages. (PNG 156 kb) Additional file 4: Figure S3. The tip cells on ISV showed protrusions without specific orientation in Thsd7a knockdown zebrafsih. Representative images showed the effects of Thsd7a knockdown by injecting Thsd7a MO2 into *Tg(fli1:EGFP)* zebrafish embryos; 5msMO2 was used as control. The morphants were then observed at 27 ~ 34hpf. In the control group, the tip cell on ISV displayed tree-shape morphology with single main protrusion (A and B). After knockdown of Thsd7a, the tip cell displayed fan-shape morphology with disorientation (C and D). Scale bar is 10 μm. (PNG 683 kb)
