# Supplementary figures and images for: Motor neuron-derived Thsd7a is essential for zebrafish vascular development via the Notch-dll4 signaling pathway
Source: J Biomed Sci. 2016 Aug 2;23:59. doi: 10.1186/s12929-016-0277-9 (PMC4971630; doi:10.1186/s12929-016-0277-9)

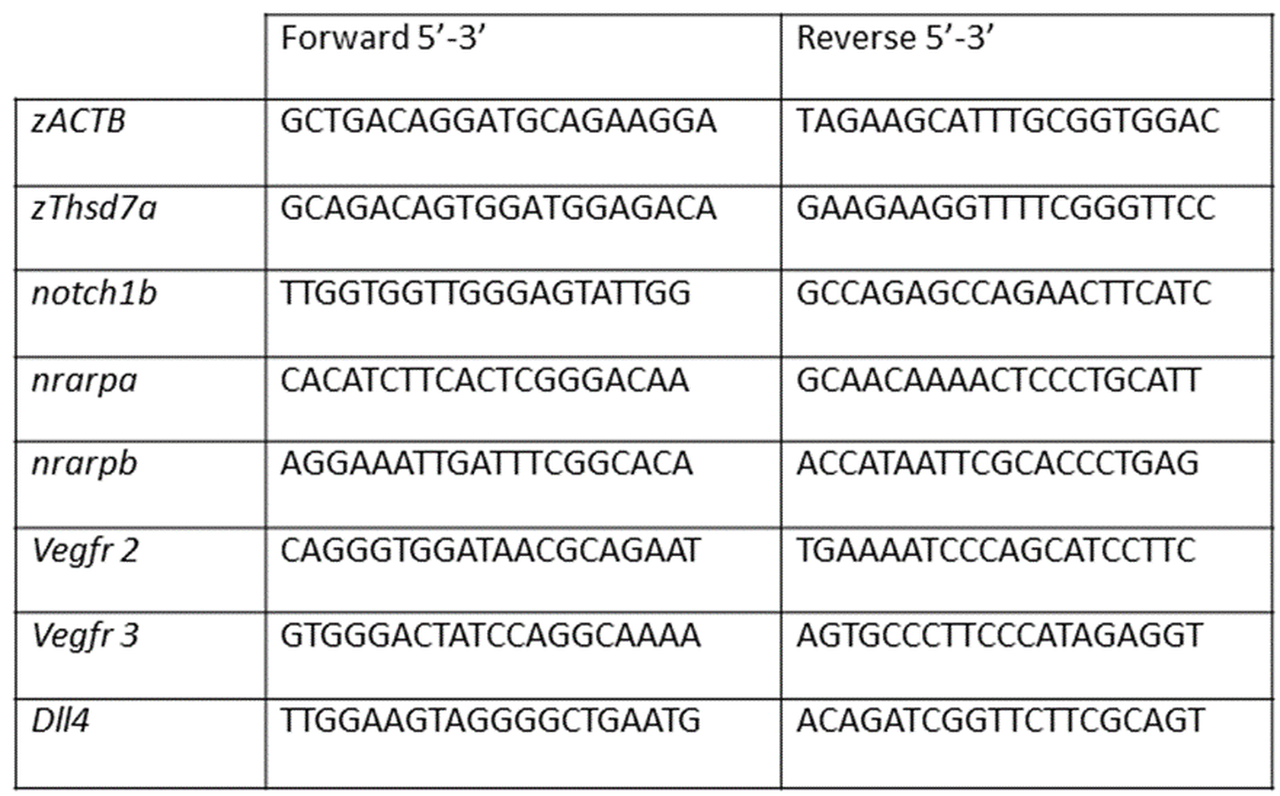

Supplement: Additional file 1: Table S1. — Primer list of Real Time-quantitative PCR analysis. (PNG 561 kb) [file 12929_2016_277_MOESM1_ESM.png]

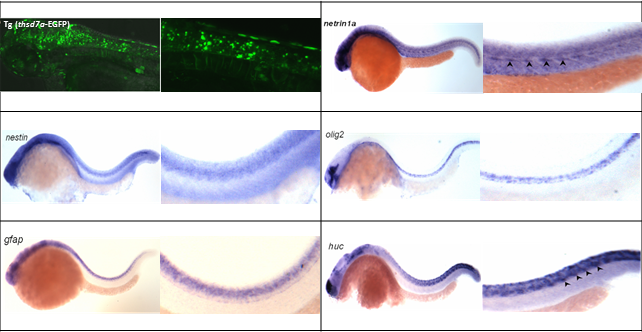

Supplement: Additional file 2: Figure S1. — Comprehensive in situ hybridization analysis of thsd7a expression to different neuronal markers. The expression of different neuronal markers include nestin (progenitor cells of nervous system)、olig2 (oligodendrocyte)、gfap (glial fibrillary acidic protein; astrocyte)、huc (Hu antigen C; mature neuron)、islet I (MiP and RoP motoneuron)、islet II (CaP motoneuron) and netrin1a (neuron tube and HMS). (PNG 210 kb) [file 12929_2016_277_MOESM2_ESM.png]

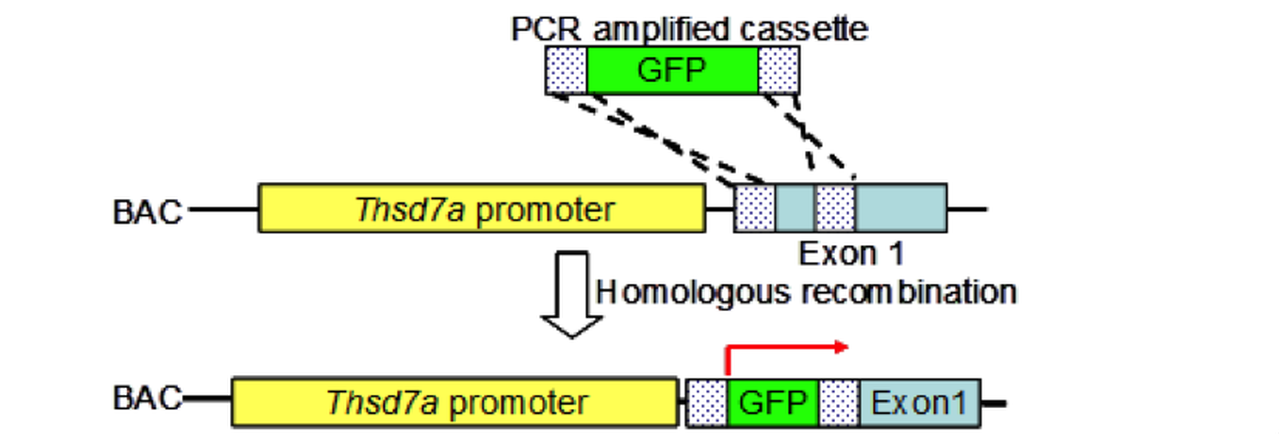

Supplement: Additional file 3: Figure S2. — The approach of construct thsd7a transgenic zebrafish. Representative image is the flow chart of making transgenic fish construct. PCR amplified cassette containing GFP was flanked by target homologous BAC sequence indicated by gray box. The target homologous site was designed to locate between the thsd7a translational start site and the first exon. BAC containing PCR product and thsd7a promoter were then electroporated into EL250 competent cells which can induce homologous recombination activity. The end product was microinjected into zebrafish embryos at one-to-two cell stages. (PNG 156 kb) [file 12929_2016_277_MOESM3_ESM.png]

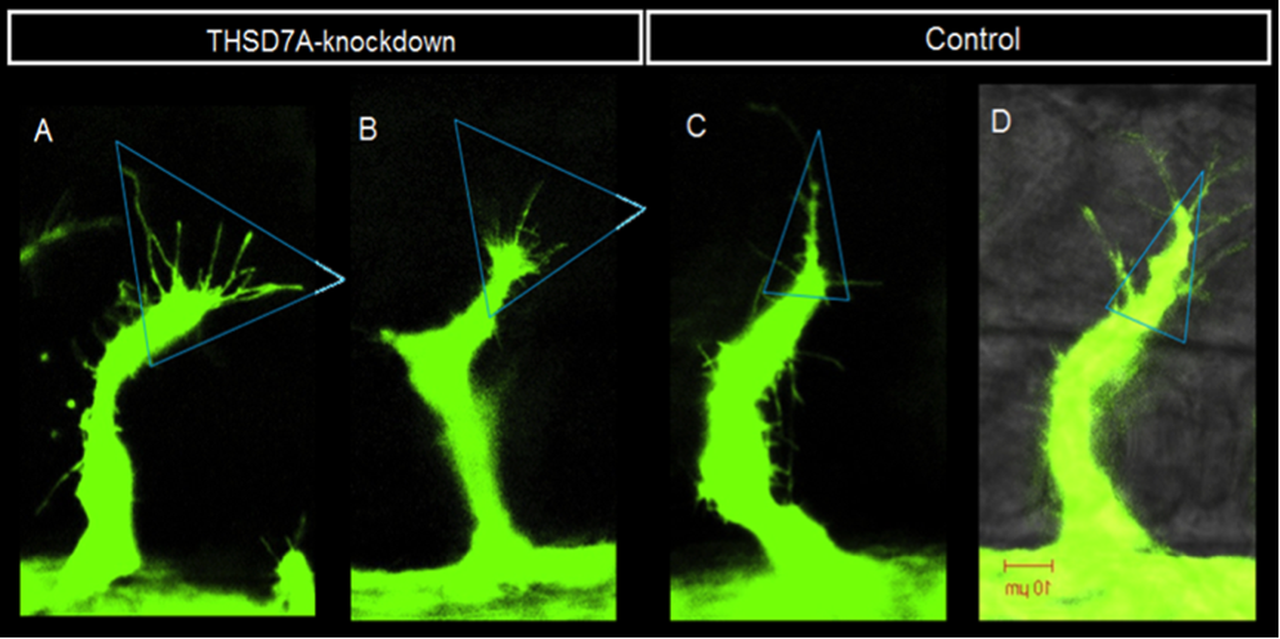

Supplement: Additional file 4: Figure S3. — The tip cells on ISV showed protrusions without specific orientation in Thsd7a knockdown zebrafsih. Representative images showed the effects of Thsd7a knockdown by injecting Thsd7a MO2 into Tg(fli1:EGFP) zebrafish embryos; 5msMO2 was used as control. The morphants were then observed at 27 ~ 34hpf. In the control group, the tip cell on ISV displayed tree-shape morphology with single main protrusion (A and B). After knockdown of Thsd7a, the tip cell displayed fan-shape morphology with disorientation (C and D). Scale bar is 10 μm. (PNG 683 kb) [file 12929_2016_277_MOESM4_ESM.png]
